# The role of overbalance pressure on mud induced alteration of sandstone rock pore system

**DOI:** 10.1038/s41598-022-12527-4

**Published:** 2022-05-19

**Authors:** Hany Gamal, Salaheldin Elkatatny, Abdulrauf Adebayo

**Affiliations:** 1 College of Petroleum Engineering and Geosciences, King Fahd University of Petroleum and Minerals, Dhahran, 31261 Saudi Arabia; 2 Center for Integrative Petroleum Research, King Fahd University of Petroleum and Minerals, Dhahran, 31261 Saudi Arabia

**Keywords:** Energy, Engineering

## Abstract

Overbalance pressure is a very critical parameter in drilling operations. It has a great impact on formation damage, depending on other downhole parameters such as temperature, time, type and composition of mud, and rock mineralogical content. The objective of this study is to determine the degree of the impact of overbalance pressure on mud–rock interaction and the resultant effects on the rock pore system. This research presents an experimental study for the interaction of a Berea Buff sandstone and barite water-based under different overbalance pressure (300, 700, and 1000 psi) under the same temperature and interaction time. The experiments involved the use of the scanning electron microscope and nuclear magnetic resonance relaxation measurements to monitor changes in the pore system of the rock samples. A modified filtration cell was used to accommodate the rock samples and mud at different overbalance pressures. The obtained results showed that the filtration properties, rock flow characteristics (rock permeability, pore throat radius, and pore system scale type) are all affected by increasing the overbalance pressure. The filtration properties increased in terms of mud cake thickness and filtrate volume by 111% and 36% respectively when the overbalance pressure was increased from 300 to 1000 psi. The total rock porosity showed a decrease from 21.6% (pre-mud interaction) to 17.6, 15.2, and 14.2% under 300, 700, and 1000 psi, respectively. The rock permeability decreased by 75% under 1000 psi overbalance pressure while pore throat radius decreased by 45%. However, the rock pore type remains on the same scale (Macro) after interaction with the mud. Statistical analysis showed that the rock porosity and permeability decreased with the overbalance pressure increase through a polynomial relationship with a high determination coefficient of 0.99. Analysis of the internal pore system by the scanning electron microscope showed that the formation damage is mainly attributed to the precipitations of mud solids as overbalance pressure is increased.

## Introduction

Drilling operation is a very critical and expensive phase in field development for petroleum production. The design of the drilling fluid and drilling operation programs require a deep study of the mud–rock interaction in order to achieve a safe and efficient drilling performance. Mud–rock interaction greatly affects wellbore stability. As a result, many drilling issues might be encountered as a result of the non-appropriate design of the mud leading to economic losses in terms of downhole equipment cost, non-productive time for the drilling operation, and remedial operations for such problems^1,2^.

The drilling fluid represents the heart of the drilling operation as it provides many functions for the drilling operation such as overbalancing the formation pressure to keep the well under control, flushing out the drilled cuttings to the surface so as to keep the well clean, lubricating and cooling the drill string during the drilling operation, and enhancing wellbore stability by forming filter cake on the wall of the drilled formations^3–5^. The composition of the drilling fluids is designed carefully to provide efficient rheological properties to achieve the required functions during the drilling operation while at the same time, protecting the drilled formation from damage. Therefore, many research studies were performed to provide new materials as additives for the mud to meet such purpose^4,6^. Formation damage is a critical problem in the petroleum industry and many studies have been conducted to provide deep analysis and technical solutions to the problem^7^.

The drilled formations are exposed to the drilling mud system during the well drilling operation which can cause severe alterations to the rock characteristics in terms of porosity, permeability, fluid flow properties, internal rock topography, pore system, elastic properties, and rock strength^8–10^. Experimental work showed that all these alterations are attributed to the rock mineralogical composition and the mud chemical activity, especially from the filtrate fluid that invades the drilled rock during the well drilling operation^11–13^.

### Overbalance pressure and mud–rock interaction

**Figure 1:**
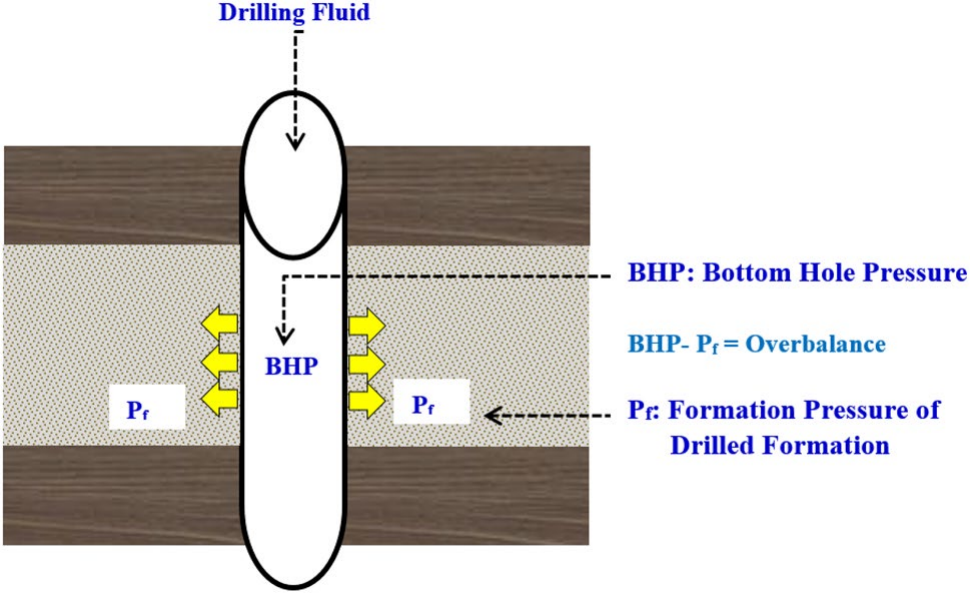
Well schematic to show the overbalance pressure during drilling.

Overbalanced drilling technique is one of the most common techniques for drilling oil and gas wells. The technique imposes bottom hole pressure that is higher than the drilled formation’s pore pressure thereby suppressing the influx of formation fluid^14,15^. The overbalance pressure is commonly known as differential pressure. In the case of a static borehole condition with no mud circulation, the overbalance pressure is mainly attained by the mud weight. In the dynamic borehole condition, the overbalance pressure is provided by both the mud pumping pressure and the mud weight^16^. As sown in Fig. 1, the overbalance pressure represents the difference between the bottom hole pressure and the drilled formation pressure. This parameter is designed by the drilling engineer, who consider many technical aspects like the drilled formation pressure, depleted zones pressure, and formation integrity^17^. The overbalance pressure affects the drilling operation performance such as the rate of penetration, pipe differential sticking, and other issues. Hence, it should be designed with deep investigations and analysis^18^.

The overbalance pressure is the main driving pressure that causes mud invasion into the drilled formation, kick-starting mud–rock interaction, and the subsequent changes in the internal pore structure of the drilled rocks. The level of the overbalance pressure is one of the parameters that affect the level of interaction between the mud and rock matrix. Many studies have been conducted to investigate mud–rock interactions involving water-based mud (WBM) that is barite-weighted and sandstone rock type under different scenarios: exposure time, clay type and content, and different weighting materials. The exposure time was studied as a key factor for the mud–rock interaction and the experimental results showed that there is a clear trend in porosity and strength reduction with increasing the exposure time^8,19^. Another study investigated the impact of exposure time on different sandstone formations such as Berea Buff and Spider, Bandera Brown, and Parker. The different rock samples were exposed to mud filtrate under the same pressure and temperature environment for a long exposure time, and the results reported that rock pore system, permeability, flow characteristics (rock permeability, pore throat radius, and pore system scale type) change to different extents based on the level of mud filtrate interaction with the clay type and content present in the rock by addressing the dominant dissolution and pore plugging phenomena for the rock samples^20^. In addition, a general strength reduction was observed within a range of 6–23% for the different sandstone samples as a result of the different clay types, clay content, and quartz-clay matrix frame^11,21^. The weighting materials were investigated for their impact on the drilled formations pore system for sandstone and carbonate rocks, and it was found that the weighting material particle size distribution is a controlling factor for the pore plugging mechanism and the filter cake characteristics during the drilling operation^22–25^.

The overbalance as a drilling parameter affects significantly the formation damage that can occur in the drilled formation even for a short time of overbalanced pressure drilling^26^. Among the literature, the depth of invasion for the drilling fluids into the drilled rock was found to increase with an increase in the overbalanced pressure up to a certain value, and then the depth of invasion slows down with a further increase in the overbalance pressure^14^. Consequently, overbalance pressure is a critical factor for mud–rock exposure and interaction, as it is the main source of the drilled formation damage, and therefore, this factor has to be studied deeply to show its impact on the rock porosity, permeability, flow characteristics, and internal pore topography. Similar experimental analysis was performed in different studies to assess the drilled rock pore system and geomechanics alterations by the impact of different factors on the rock-mud interaction for the downhole drilling operation as the extended exposure time^8,19^, different clay content and type 11,20 and different weighting agents for the drilling fluids 22,24. However, the current study presents extensive research to assess the role of overbalance pressure on the mud–rock interaction of sandstone rock. The novel contributions from the current research include providing an integrated experimental workflow for assessing the changes in the pore system of rocks with the use of X-ray diffractions (XRD) and scanning electron microscopes (SEM) to study the rock and pore system alterations, nuclear magnetic resonance (NMR) technique to evaluate the rock pore size distribution. The study employed a modified aging cell for the filter press system to accommodate the rock sample for the mud–rock interaction under the pressure, temperature, time environment to simulate the downhole conditions for mud–rock interaction during the drilling operation.

## Study materials and experimental methods

Sandstone rock samples from Berea Buff type was used to represent the rock type with exposure for the drilling fluid of barite weighted WBM under the same conditions of temperature (200 °F), time (30 min of filtration as per the standard procedures^27^), while the overbalance/differential pressure ranged from 300, 700, and 1000 psi, to investigate the role of overbalance pressure as a factor for the mud–rock interaction process.

### Experimental design

**Figure 2:**
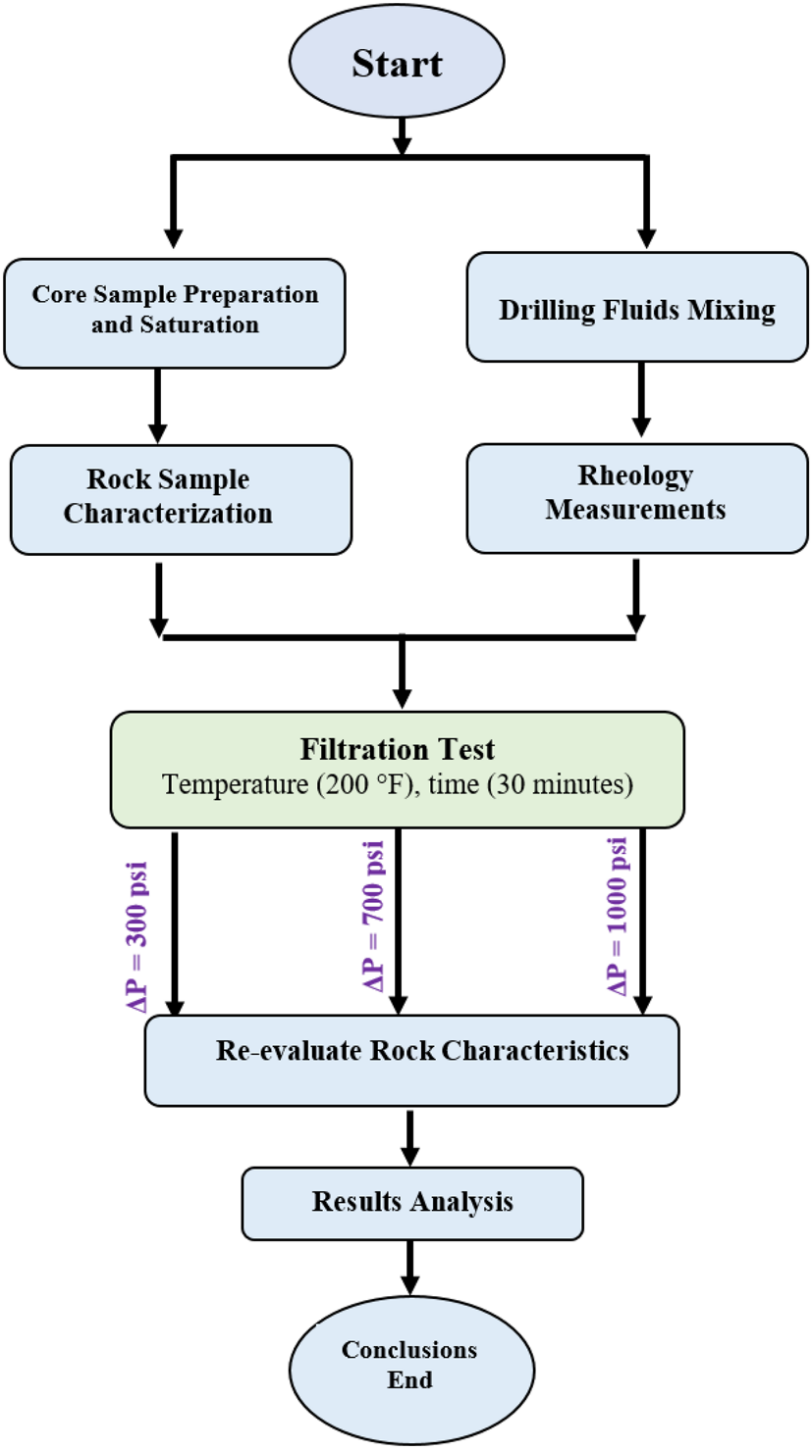
Experimental design layout.

**Figure 3:**
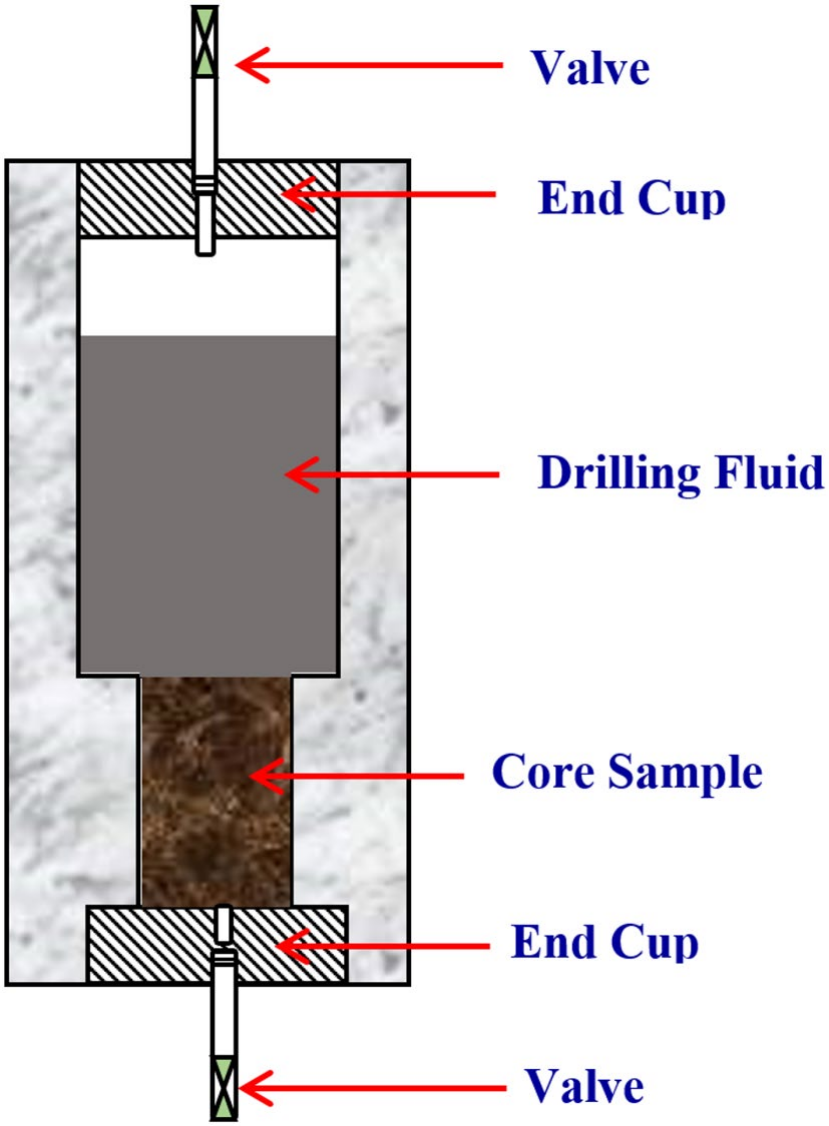
Schematic for the modified cell for the filtration test.

Figure 2 illustrates the experimental design layout for the current study to achieve the proposed objective of studying the role of overbalance pressure on mud–rock interaction under the downhole environment during the drilling operation. The rock samples (Berea Buff) were prepared and cut into cylindrical shapes (2 inches length by 1.5 inches diameter) and saturated with 3 wt.% potassium chloride (KCl) to provide clay stability and inhibit the clay swelling^28^. An integrated rock characterization was performed for the rock samples as a mineralogical composition by XRD analysis, SEM, and NMR spectrometry. This phase was followed by the filtration test, where the mud and rock samples were accommodated in the modified cell for the filtration test as shown in Fig. 3. The mud–rock interaction was executed under the designed environment of temperature (200 °F), time (30 min), and different scenarios of overbalanced pressure (300, 700, and 1000 psi). During the filtration test, the mud–rock interaction starts, and the filtration properties for the test were recorded as filtrated volume and the filter cake thickness was recorded after the filtration test. The rock samples' properties were evaluated after the mud–rock exposure to compare with the initial condition (saturated samples).

The mineralogical composition for the sandstone rock samples was determined using XRD which showed that quartz contributed 91 wt.%, microcline constituted 4 wt.%, clay content of 5 wt.% (which includes kaolinite, smectite, and muscovite with 3, 1, and 1 wt.% respectively). The quartz clay frame is very critical to the mud interaction besides the clay content and type^29,30^.

The drilling fluid that was used in this study is WBM and it was prepared as per the mud formulation in Table 1. The formulation contains water as the base fluid for the WBM drilling fluid, polymer (XC) and bentonite as mud viscosity control, starch was added to control the fluid loss, calcium carbonates with a size of 50 microns (D_50_) as a bridging agent, and barite was used to control the mud weight. The mud rheological properties for the formulation was measured at 80 °F temperature as the normal procedure on the rig site and the recorded values were listed in Table 2 as the mud weight is 12.25 ppg, and pH of 9.5 which is considered a good range for the mud pH that will greatly enhance the mud filtration properties^31^. The mud viscosity was 13 cP, while yield point, initial, and 10-min gel strength showed 63, 11, 21 lb/100 ft^2^.

**Table 1:** WBM formulation for the study.

Additives	Quantity, g	Function
Water	290	Base fluid
Defoamer	0.08	Anti-foam agent
XC-polymer	1.5	Viscosity control
Bentonite	4	Viscosity control
Starch	6	Fluid loss agent
Potassium chloride	20	Clay swelling inhibition
Potassium hydroxide	0.3	pH control
Calcium carbonates	5	Bridging agent
Barite	200	Mud weight control

**Table 2:** WBM rheological properties measurements at 80 °F.

Mud property	Value
Density, ppg	12.25
pH	9.5
Plastic viscosity, cP	13
Yield point, lb/100 ft^2^	63
Initial gel strength (10 s), lb/100 ft^2^	11
10 min-gel strength, lb/100 ft^2^	21

### Pore system characterization

The rock pore system was characterized by utilizing the spectrometry analysis for the scanning electron microscope (associated with the feature of energy-dispersive X-ray) and nuclear magnetic resonance was introduced as rock characterization tools in the petroleum industry and provided successful research studies^32–34^.

SEM analysis provides information about the internal topography of rock samples in terms of pore plugging due to swelling or slides precipitations, the opening of pores caused by rock dissolution after mud–rock interaction^35^, hence, the integrity of rock pore system can be studied using SEM to provide an indication changes in pore system after the mud–rock interaction at different levels of overbalance pressure^36^.

NMR provides a measurement of the transverse relaxation time ($${T}_{2})$$ of hydrogen laden fluid saturating the pores of rock samples, where $${T}_{2}$$ value is proportional to the radius of the rock pore throat^37^. Hence, the pore size distribution of rock samples can be plotted, analyzed, and compared between pre and post-mud interaction phases to assess changes in the pore system at different overbalance pressures^38,39^. NMR results can be performed to have the rock porous system flow characteristics (rock permeability, pore throat radius, and pore system scale type)^40–42^ as follow:

1 $$k= C {T}_{2}^{2}\;{\Phi}^{4}$$

2 $${\mathrm{Log\;r}}_{35}= 0.732 + 0.588\;Log\;K - 0.864\;Log\;\Phi$$

where k is the permeability of rock sample in mD, T_2_ in milliseconds, and $\Phi$ is the porosity of rock samples (fraction). C is a statistical parameter from the experimental data. r_35_ is the pore throat radius equivalent to 35% mercury saturation in µm.

## Results and discussion

This section illustrates, analyzes, and discusses the results obtained from the experimental work. The results show the changes that occurred in the rock samples pore system due to the role of overbalance pressure on the interaction between the rock samples and mud.

### Filtration properties

The filtration test was performed at three levels of overbalance pressure (300, 700, and 1000 psi), where, all other test parameters were fixed to study and assess the role of overbalance pressure. The results are tabulated in Table 3 and show that the total filtrate volume (recorded in 30 min filtration test) increases with the overbalance pressure value, where the total mud filtrates were 5.5 cm^3^, 6.6 cm^3^, and 7.5 cm^3^ under 300, 700 and 1000 psi overbalance pressure, respectively. The results reveal that there is an 18% increase in the total recorded filtrate volume after increasing the overbalance pressure from 300 to 700 psi, while this percentage was a 36% increase in the total filtrate volume after increasing the overbalance pressure from 300 to 1000 psi. On the other side, the recorded thickness for the mud cake was also increased with the applied overbalance pressure as it recorded 1.51 mm under 300 psi, 2.29 mm (52% increase) with 700 psi, and 3.18 mm (111% increase) with 1000 psi overbalance pressure level. The measured filtration test results prove the role of overbalance pressure as a downhole parameter for the mud–rock interaction as the filtration properties (in terms of mud filtrate and filter cake thickness) increase with increasing the overbalance pressure.

**Table 3:** Filtration properties with the overbalance pressure.

Filtration property	Under 300 psi	Under 700 psi	Under 1000 psi
Total filtrate volume, cm^3^	5.5	6.6	7.5
Mud cake thickness, mm	1.51	2.29	3.18

### Sandstone formation damage

**Figure 4:**
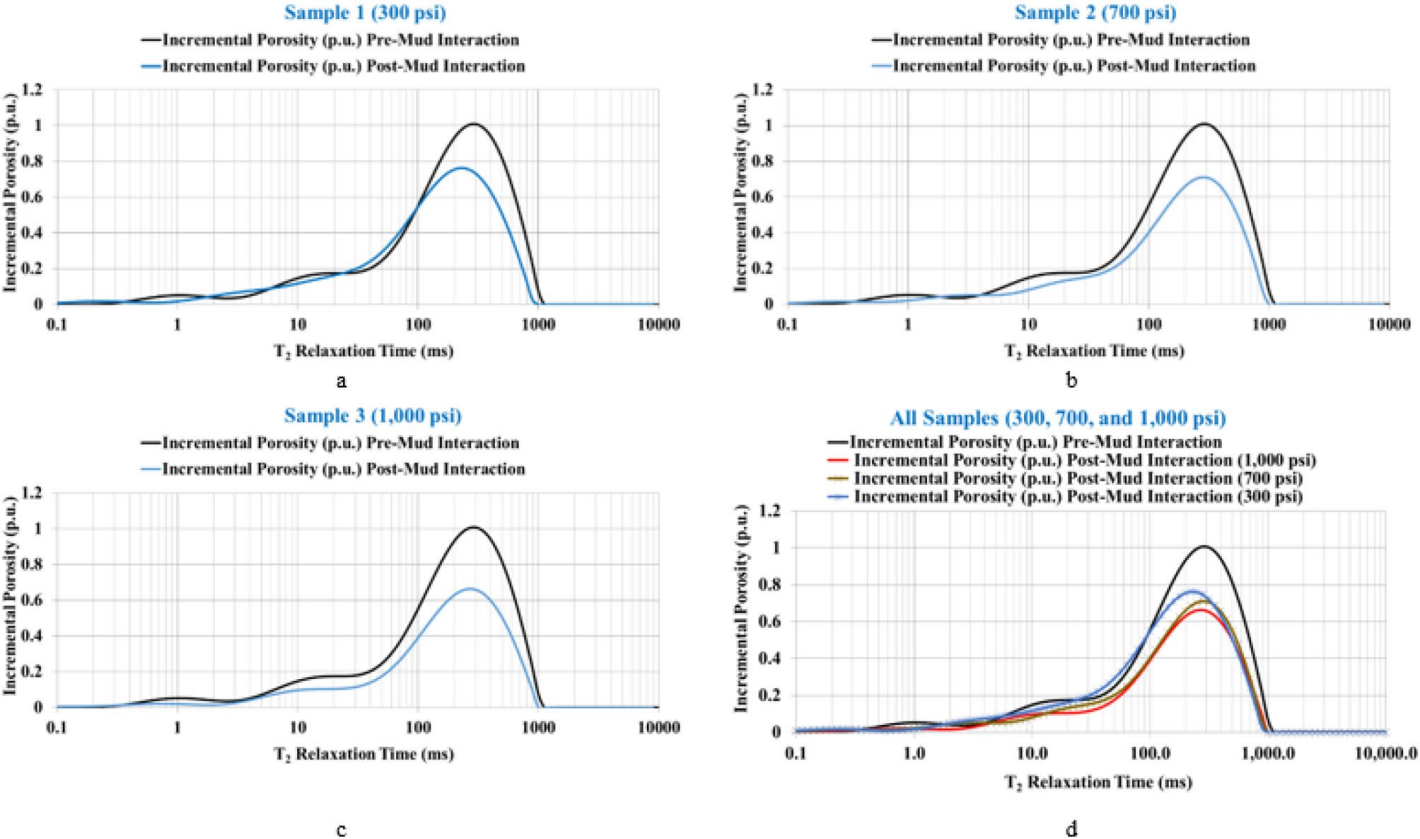
The incremental porosity plots with the overbalance pressure. (**a**) Under 300 psi overbalance. (**b**) Under 700 psi overbalance. (**c**) Under 1000 psi overbalance. (**d**) all samples.

The sandstone rock samples were evaluated in terms of rock porosity, distribution of pore size permeability, pore throat radius, and pore system type. Studying these parameters provides important information about the damage level for the rock samples with an increase in the overbalance pressure for the mud–rock interaction. Figure 4 shows the NMR incremental porosity of the rock samples in the initially saturated case (pre-mud interaction) and after interaction with the mud (post-mud interaction) along with the T_2_ range that represents the equivalent pore radius for the sandstone samples. The results showed an obvious reduction in the incremental porosity profile under 300 psi overbalance pressure (Fig. 4a) and further reduction with increasing overbalance pressure (700 and 1000 psi) for samples 2 and 3 (Fig. 4b,c) and all the samples profile are shown in Fig. 4d. The plots show that pore plugging can be attributed to the mud solids precipitations after mud–rock interaction.

**Figure 5:**
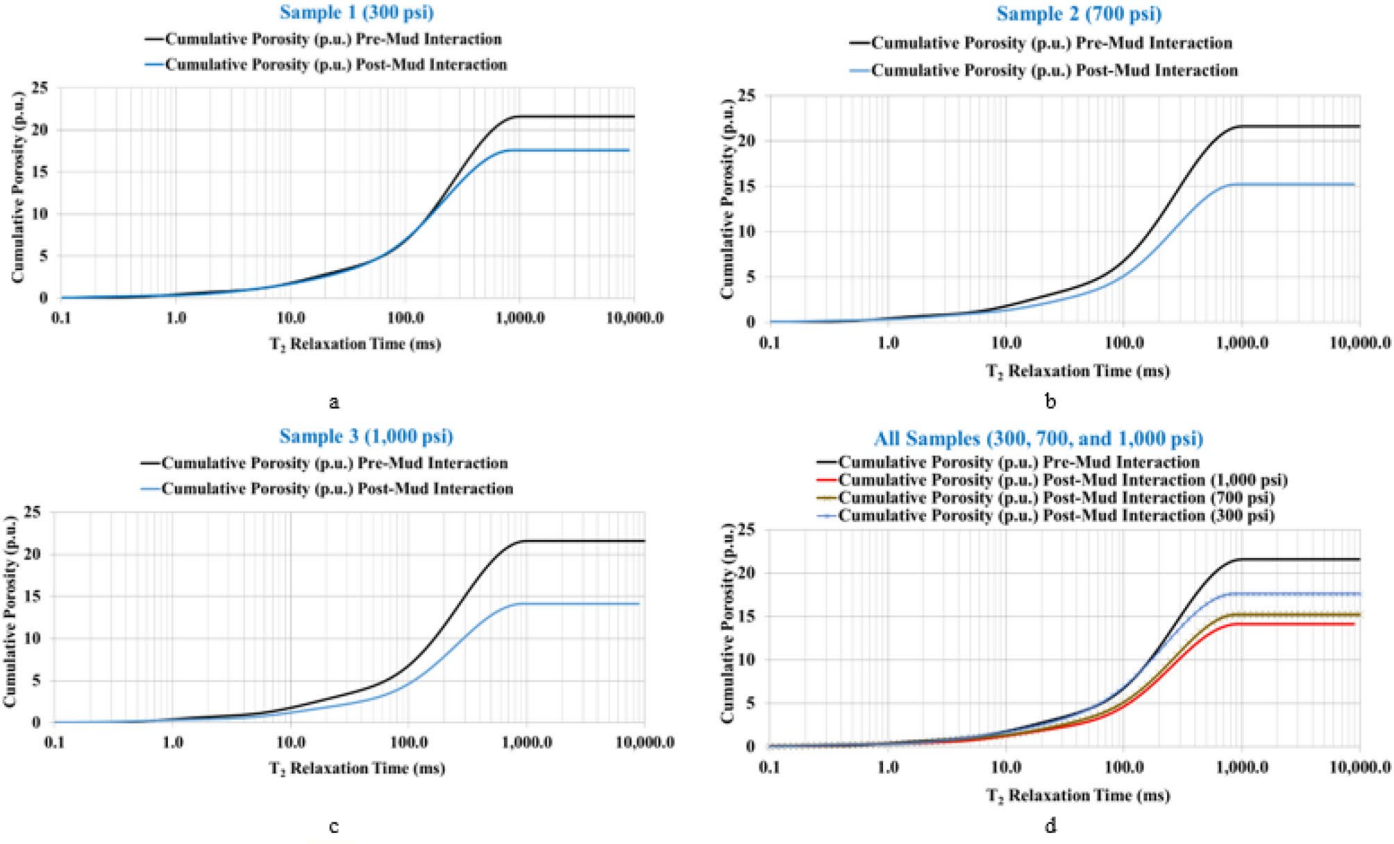
The cumulative porosity plots with the overbalance pressure. (**a**) Under 300 psi overbalance. (**b**) Under 700 psi overbalance. (**c**) Under 1000 psi overbalance. (**d**) all samples.

In addition, plotting the profiles of cumulative porosity for the sandstone samples over the different ranges of overbalance pressure illustrates the total porosity reduction for the three samples (Fig. 5). The plots show that the total porosity decreased from 21.6% to record 17.6% under 300 psi overbalance pressure (Fig. 5a), decreased to 15.2% under 700 psi overbalance (Fig. 5b), and reached 14.2% total porosity under 1000 psi overbalance (Fig. 5c) that concludes the reduction of total rock porosity (from 21.6 to 14.2%) with increasing the overbalance pressure from 300 to 1000 psi. Another important observation about the degree of extent of the formation damage to the internal pore system is that the small pores are affected by increasing the overbalance pressure; as by applying only 300 psi overbalance, the large size pore system is affected (T_2_ greater than or equal to 200 ms) as clearly shown in Fig. 5a. Increasing the overbalance pressure to 700 psi presented formation damage to the pore system that has T_2_ greater than or equal to 8 ms (Fig. 5b), and this impact increased to affect the pore size with T_2_ greater than 2 ms by extending the overbalance pressure to 1000 psi as clear from Fig. 5c. The comparative plots for the three samples are shown in Fig. 5d which shows clearly the decreasing behavior of the cumulative porosity with increasing the overbalance pressure level.

The rock permeability was determined for the pre- and post-mud interaction under different overbalance pressures and the results showed that the permeability decreased with an increase in the overbalance pressure. The permeability decreased from its initial value of 170 mD to 105 mD under 300 psi overbalance representing a 38% permeability reduction, and this impact increased while increasing the overbalance pressure to 700 and 1000 psi to cause permeability reduction to 56 and 43 mD respectively.

Furthermore, the pore throat radius that is equivalent to 35% mercury saturation was determined to study the impact of the mud–rock interaction on the pore system type, and the results showed that increasing the overbalance pressure from 300 to 1000 psi caused a reduction behavior for the R_35_ from 9 to 5 µm, while the size of the pore system is still in the macro-scale even after the mud–rock interaction. This is attributed to the good storage and flow characteristics of the sandstone samples, which makes the impact of overbalance pressure on the mud–rock interaction minimal and not enough to reduce the pore system type to meso- or micro-scale. Table 4 summarises the results for the pore system characteristics for the pre-mud case and with increasing the overbalance pressure scenarios.

**Table 4:** Pore system characteristics with increasing overbalance pressure.

Case	Total porosity, %	Porosity reduction, %	K, mD	K reduction, %	R_35_, µm	Pore system type
Pre-mud Interaction	21.6		170		9.0	Macro
300 psi Overbalance	17.6	19	105	38	7.0	Macro
700 psi Overbalance	15.2	30	56	67	5.5	Macro
1000 psi Overbalance	14.2	34	43	75	5.0	Macro

### Damage mechanism

**Figure 6:**
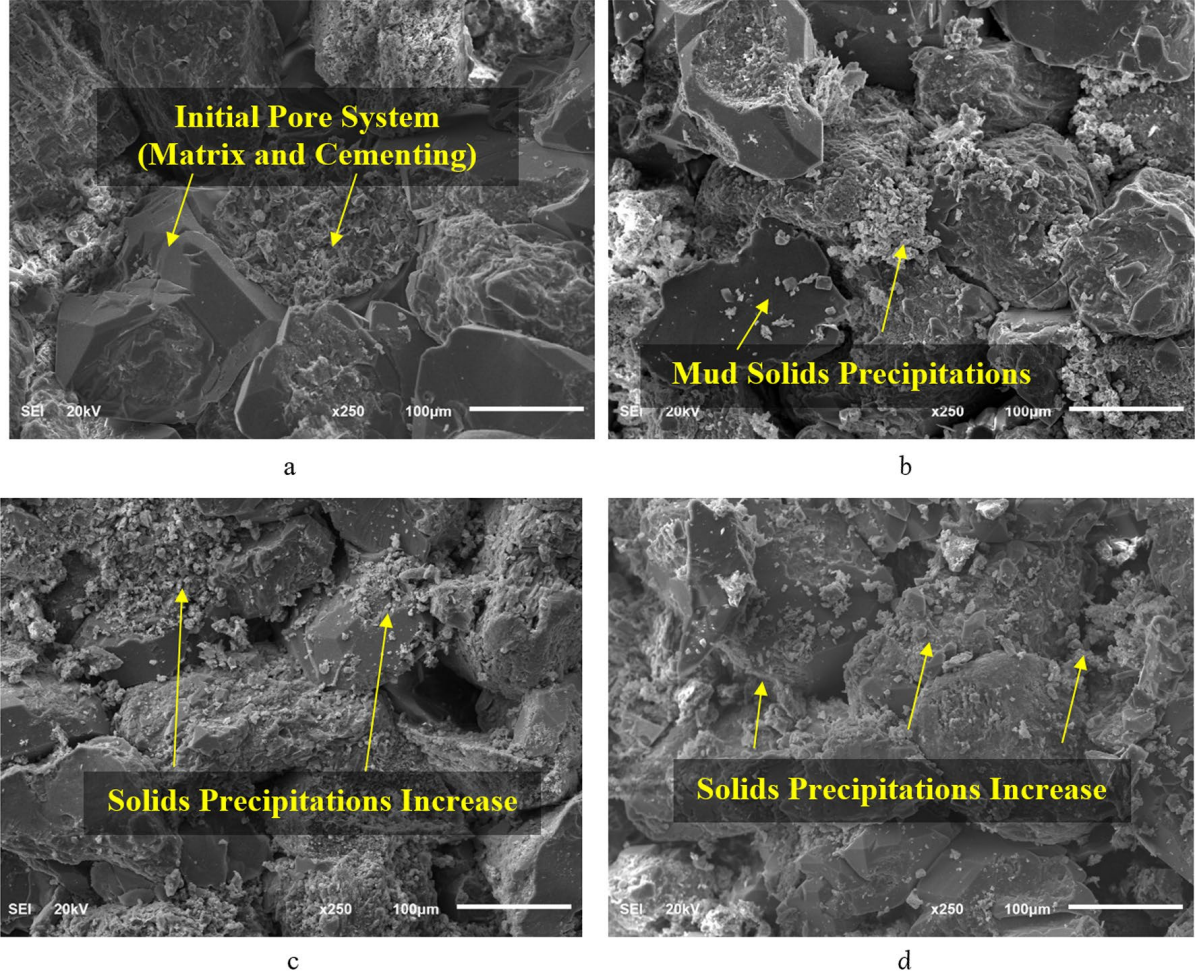
Internal pore system changes with the overbalance pressure. (**a**) Initial saturated core. (**b**) Under 300 psi overbalance. (**c**) Under 700 psi overbalance. (**d**) Under 1000 psi overbalance.

Studying the internal pore system topography can assist in analyzing the alterations of the pore system of the sandstone rock samples from the initial condition of the sample (pre-mud interaction) and post-mud interaction after imposing different levels of overbalance pressure. SEM analysis (Fig. 6) for the sandstone samples illustrates that the pore system showed pore plugging impact due to precipitation of the mud solids during the filtration test. This confirms the results obtained for the pore system porosity reduction. The initial state of the rock matrix and cementing material are clearly shown in Fig. 6a. Figure 6b shows precipitation of mud solids in the pores after an overbalance pressure of 300 psi. while Fig. 6c,d show mud precipitates after an overbalance pressure of 700 psi and 1000 psi respectively. The mud solids precipitations increased due to the overbalance pressure increase because of the driving force of the overbalance pressure that forces solids and filtrate to invade the rock pore system.

**Figure 7:**
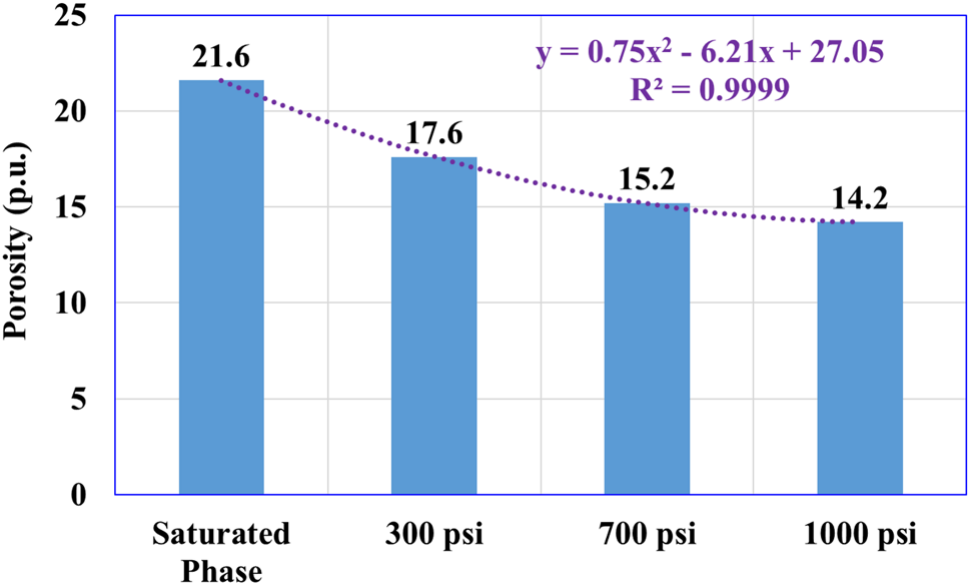
Porosity reduction versus the applied overbalance pressure.

**Figure 8:**
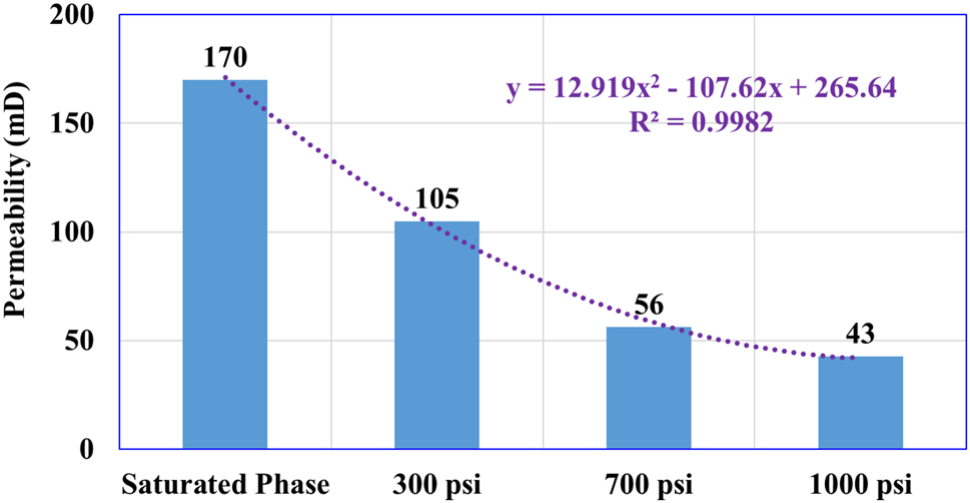
Permeability reduction versus the applied overbalance pressure.

Further statistical analysis was performed for the obtained results to relate the porosity and permeability of the damaged rock samples to the applied overbalance pressure and showed that there is a clear strong relationship with a high degree of determination coefficient (R^2^) that is close to 1 as shown from Figs. 7 and 8. The porosity and permeability values can be estimated from the polynomial relationship (order 2) as follow:

3 $$ \Phi = 0.{75}^{*}\left( {\Delta {\text{P}}} \right)^{{2}} - {6}.{21}^{*}\left( {\Delta {\text{P}}} \right)  + {27}.0{5} $$

4 $$ {\text{K = 12}}.{919}^{*}\left( {\Delta {\text{P}}} \right)^{{2}} - {1}0{7}.{62}^{*}\left( {\Delta {\text{P}}} \right) + {265}.{64} $$

where $\Phi$ is in porosity unit, ΔP is the overbalance pressure in psi, K in mD.

The obtained correlations have the limitations of the experimental conditions when talking about the general application in terms of the implemented rock type (Berea Buff sandstone), mud system (barite-weighted water-based mud), and the interaction environment parameters of overbalance pressure (300 upto1000 psi), temperature (200 °F), time (30 min). General application beyond the aforementioned conditions has to be tested and validated through experimental design.

The developed correlations show the statistical relationship in addition to the physical base for relating the porosity and permeability reduction to the extended overbalance pressure as the obtained results from the extensive experimental work and results` analysis showed that the degree of formation damage is highly impacted by the role of overbalance pressure on the mud–rock interaction as increasing the overbalance pressure will increase the formation damage for the sandstone rock samples in terms of rock porosity, distribution of pore size, permeability, and pore throat radius, and pore system scale type.

## Conclusions

The current research presented an experimental workflow to assess the role of overbalance pressure on mud–rock interaction during drilling operations. Barite-weighted WBM and sandstone samples (Berea Buff) were employed to determine the formation damage that occurred under different levels of overbalance pressure (300, 700, and 1000 psi) while keeping constant, all other mud–rock environments (temperature, 200 °F) and time (30 min). The following conclusions are derived from this study:

- The mud filtration properties were increased with an increase in the overbalance pressure as the mud cake thickness increased from 1.51 to 3.18 mm (111% increase), while the total recorded filtrate volume increased from 5.5 to 7.5 cm^3^ (36% increase).
- Overbalance pressure affects rock porosity, where increasing the overbalance pressure from 300 to 1000 psi causes total porosity reduction from 21.6 to 14.2% with a 34% reduction for the rock porosity after the mud–rock exposure.
- Rock permeability decreased from 170 mD (initial condition) under increasing the overbalance pressure from 300, 700, and 1000 psi to record 105, 56, 43 mD respectively.
- Statistical analysis showed that the rock porosity and permeability decreased with the overbalance pressure increasing through a polynomial relationship with a high determination coefficient of 0.99.
- The pore throat radius (equivalent to 35% mercury saturation) for the sandstone samples showed a reduction trend (after increasing the overbalance pressure) to record 5 µm under 1000 psi overbalance from the initial condition (pre-mud interaction) of 9 µm. However, the rock pore system remains the same pore-scale (Macro type) after the mud–rock interaction with increasing the overbalance pressure.
- The SEM analysis confirmed that the obtained formation damage represented by the pore plugging mechanism is because of the mud solids precipitations, which increased with increasing overbalance pressure.

## Data Availability

The used data are available in this manuscript. No more data were used in this study.
